# Deep-learning-based stock market prediction incorporating ESG sentiment and technical indicators

**DOI:** 10.1038/s41598-024-61106-2

**Published:** 2024-05-04

**Authors:** Haein Lee, Jang Hyun Kim, Hae Sun Jung

**Affiliations:** 1 Department of Applied Artificial Intelligence/Department of Human–Artificial Intelligence Interaction, Sungkyunkwan University, Seoul, 03063 Republic of Korea; 2 Department of Interaction Science/Department of Human–Artificial Intelligence Interaction, Sungkyunkwan University, Seoul, 03063 Republic of Korea; 3 Department of Applied Artificial Intelligence, Sungkyunkwan University, Seoul, 03063 Republic of Korea

**Keywords:** ESG, Natural language processing (NLP), Time series prediction, Deep learning, Computer science, Computational science

## Abstract

As sustainability emerges as a crucial factor in the development of modern enterprises, integrating environmental, social, and governance (ESG) information into financial assessments has become essential. ESG indicators serve as important metrics in evaluating a company’s sustainable practices and governance effectiveness, influencing investor trust and future growth potential, ultimately affecting stock prices. This study proposes an innovative approach that combines ESG sentiment index extracted from news with technical indicators to predict the S&P 500 index. By utilizing a deep learning model and exploring optimal window sizes, the study explores the best model through mean absolute percentage error (MAPE) as an evaluation metric. Additionally, an ablation test clarifies the influence of ESG and its causality with the S&P 500 index. The experimental results demonstrate improved predictive accuracy when considering ESG sentiment compared to relying solely on technical indicators or historical data. This comprehensive methodology enhances the advantage of stock price prediction by integrating technical indicators, which consider short-term fluctuations, with ESG information, providing long-term effects. Furthermore, it offers valuable insights for investors and financial market experts, validating the necessity to consider ESG for financial assets and introducing a new perspective to develop investment strategies and decision-making processes.

## Introduction

Sustainability represents a crucial global trend in shaping the progress of modern enterprises. Given the increasing emphasis on sustainable practices, integrating environmental, social, and governance (ESG) indicators to assess a company’s performance has become indispensable^1,2^. ESG indicators measure a company’s ESG performance, providing valuable insights into operational practices^3^. When a company embraces sustainable business model, fulfills social responsibilities, and upholds effective governance, investors can place a high degree of trust in the company and evaluate its future growth potential positively^4^. This positive perception of future growth potential can translate into stock price increases for the company. Consequently, ESG indicators significantly influence decision-making of investors^5^, thereby encouraging companies to proactively adopt sustainable practices and improve their ESG metrics while also demonstrating that sustainable business strategies offer advantages to both investors and companies.

The interconnection between sustainability and ESG metrics and their impact on investor choices highlights the increasing importance of incorporating ESG criteria into financial assessments, bridging corporate responsibility and investment strategies. Moreover, as financial markets evolve to accommodate these shifts, combining ESG indices to predict the S&P 500 index can be an innovative approach that considers the situation where ESG factors are increasingly affecting investment choices^6^. The reason for using the S&P 500 as the target for prediction here is because this index is considered an important indicator representing the economic situation of the United States and the health status of its companies, and it is sometimes used to reflect trends in the global stock market^7,8^. Investors are showing a growing interest in understanding how a company's ESG performance impacts its stock price, underscoring the rising significance of ESG metrics in the financial domain.^9,10^.

In addition to ESG indicators, technical indicators play an important role in financial analysis^11^. These indicators are calculated using historical data such as past price movements and trading volume of specific assets, and it is crucial for considering past price patterns or trends and predicting future movements^12^. Given the fundamental importance of technical indicators in financial analysis, their integration with ESG metrics shows significant promise, as exemplified by the comprehensive approach suggested in this study.

Therefore, research that incorporates ESG information with technical indicators for predicting S&P 500 index can provide innovative methodologies and valuable insights for investors and financial market experts. This comprehensive approach can assist in forecasting stock behavior and is anticipated to bring a fresh dimension to formulating investment strategies and decision-making. For the experiment, the authors utilized 18 technical features to predict the closing value of the S&P 500 index. Additionally, the ESG-related sentiment information obtained through the sentiment analysis of LexisNexis news data were integrated with technical indicators and applied to a regression model to predict future value of S&P 500 index and the mean absolute percentage error (MAPE) was used as an evaluation metric. As a result, the authors obtained optimal results by validating across a range of window sizes and parameters. In addition, conducting an ablation test verified that considering the ESG sentiment information is more effective than solely using technical indicators or historical price data.

## Related works

### Previous research on stock price prediction considering news text sentiment analysis

Research conducted over the past few years has devoted significant effort for investigating the correlation between news sentiment and stock prices. Zubair and Cios^13^ collected news from Reuters over a period of seven years and conducted sentiment analysis using the Harvard General Inquirer on a daily basis. The authors utilized the Kalman filter for smoothing and revealed a strong correlation between the S&P 500 index and sentiment scores. Khedr and Yaseen^14^ derived a sentiment index from news articles concerning company dividends, stock dividends, and stock mergers. Employing numerical data attributes such as open, close, high, and low prices, the authors implemented a two-stage methodology incorporating naïve Bayes for sentiment analysis, achieving an 89.80% accuracy rate in stock prediction. Li and Pan^15^ modified both news and stock data to detect forthcoming stock market trends and introduced an ensemble method, resulting in a 57.55% decrease in mean squared error (MSE) compared to baseline models.

Ultimately, these studies emphasize the dynamic interactions among news data, sentiment analysis, and stock price prediction, showcasing the various approaches and significant progress achieved in the field.

### Exploring the influence of ESG on the stock performance

Prior research has demonstrated that ESG factors affect both the valuation of corporations and the favorable perception of companies, potentially leading to a positive impact on their stock prices^16,17^. Alareeni and Hamdan^6^ conducted a statistical analysis of ESG disclosures and corporate performance metrics over 4869 days for companies in the S&P 500 index between 2009 and 2018, revealing that ESG disclosures positively influence corporate performance metrics. Minutolo et al.^18^ examined corporate performance for 467 companies included in the S&P 500 between 2009 and 2015, found that ESG has a positive impact on Tobin's q and Return on Assets (ROA) across all models, with varying effects based on company size. Gillan et al.^19^ examined ESG and corporate social responsibility (CSR), focusing on corporate finance. The study highlights that ESG and CSR activities are closely associated with a company’s market characteristics as well as with its risks, performance, and value. Zheng et al.^20^ reported that ESG performance significantly enhanced the corporate value of listed companies, particularly through the mediating roles of media attention and analyst coverage. ESG factors are crucial risk factors for firms. Companies endeavor to take socially responsible actions to consider ESG and reputational risk. Stellner et al.^21^ explored whether excellent CSR performance reduces credit risk, finding that a country’s ESG performance alleviates the relationship between corporate social performance and credit risk. Additionally, this comprehensive examination underscores the pivotal role of ESG evaluations in shaping a company’s stock price and overall corporate value.

With this recognition, companies are striving to adopt strategies that strengthen their social responsibility and environmental impact by integrating ESG information, and these efforts can be reflected to the public through news articles. Furthermore, when exposed to the public, this information can ultimately influence corporate value and, consequently, stock prices.

### Leveraging technical indicators in asset price prediction

Researchers have been devising and considering various technical indicators in attempts to predict asset performance. Xu and Keselj^22^ gathered stock data for 11 industries, along with financial tweets. To predict the stock data effectively, the authors calculated technical indicators, including AD, ADX, EMA, KAMA, MA, MACD, RSI, PSAR, and SMA (Table 1). Hoseinzade and Haratizadeh^23^ improved the futures prediction performance of a market using feature extraction. Specifically, they designed an architecture that employing technical indicators such as MOM, ROC, and EMA. In addition, the authors incorporated historical data, resulting in a 9% enhancement in F-measure performance. Assis et al.^24^ calculated technical indicators utilizing technical analysis library (TA-Lib) and employed restricted Boltzmann machines for capturing latent features and analyzed financial time series data through support vector machines. As a result, the experimental result demonstrated better accuracy compared to not using technical indicators. Jung et al.^25^ combined technical and sentiment indicators to predict Bitcoin price trends using the RSI, SMA, EMA, MACD, signal, Stochastic RSI, and Stochastic Oscillator indices. As a result, considering 11 technical indicators was found to be effective, with XGBoost exhibiting a prediction performance of 90.57%.

**Table 1:** Descriptions of technical indicators employed in previous research.

Indicator	Description
Simple Moving Average (SMA)	Provides a smoothing effect on price data over a designated time frame
Exponential Moving Average (EMA)	Provides a smoother perspective of price trends, emphasizing recent data
Chaikin Accumulation/Distribution Line (AD)	Measures cumulative buying and selling pressure for predicting price trends
Average Directional Movement Index (ADX)	Mean directional movement indicator
Kaufman Adaptive Moving Average (KAMA)	Adapts to changing market conditions, aiding in identifying optimal entry and exit points
Moving Average Convergence/Divergence (MACD)	Convergence and divergence of moving averages
Relative Strength Index (RSI)	Evaluates asset’s overbought or oversold conditions, guiding potential reversals
Parabolic Stop and Reverse (PSAR)	Offers dynamic stop-loss levels, crucial for risk management
Momentum (MOM)	Measure the rate of change
Rate of Change (ROC)	Measure the percentage change in price from a previous period to the current period
Signal	Provide partial visual smoothing of technical indicators and detect trend reversals and crossovers
Stochastic RSI	Combination of the RSI and Stochastic indicator
Stochastic Oscillator	Relative position of prices over a given period

In summary, stock predictions span various domains, prompting researchers to explore diverse variables for accurate forecasts. Approaches that calculate technical indicators from stock data and utilize sentiment indicators have significantly improved prediction accuracy, providing a solid foundation for obtaining comprehensive financial knowledge and making informed decisions.

## Method

**Figure 1:**
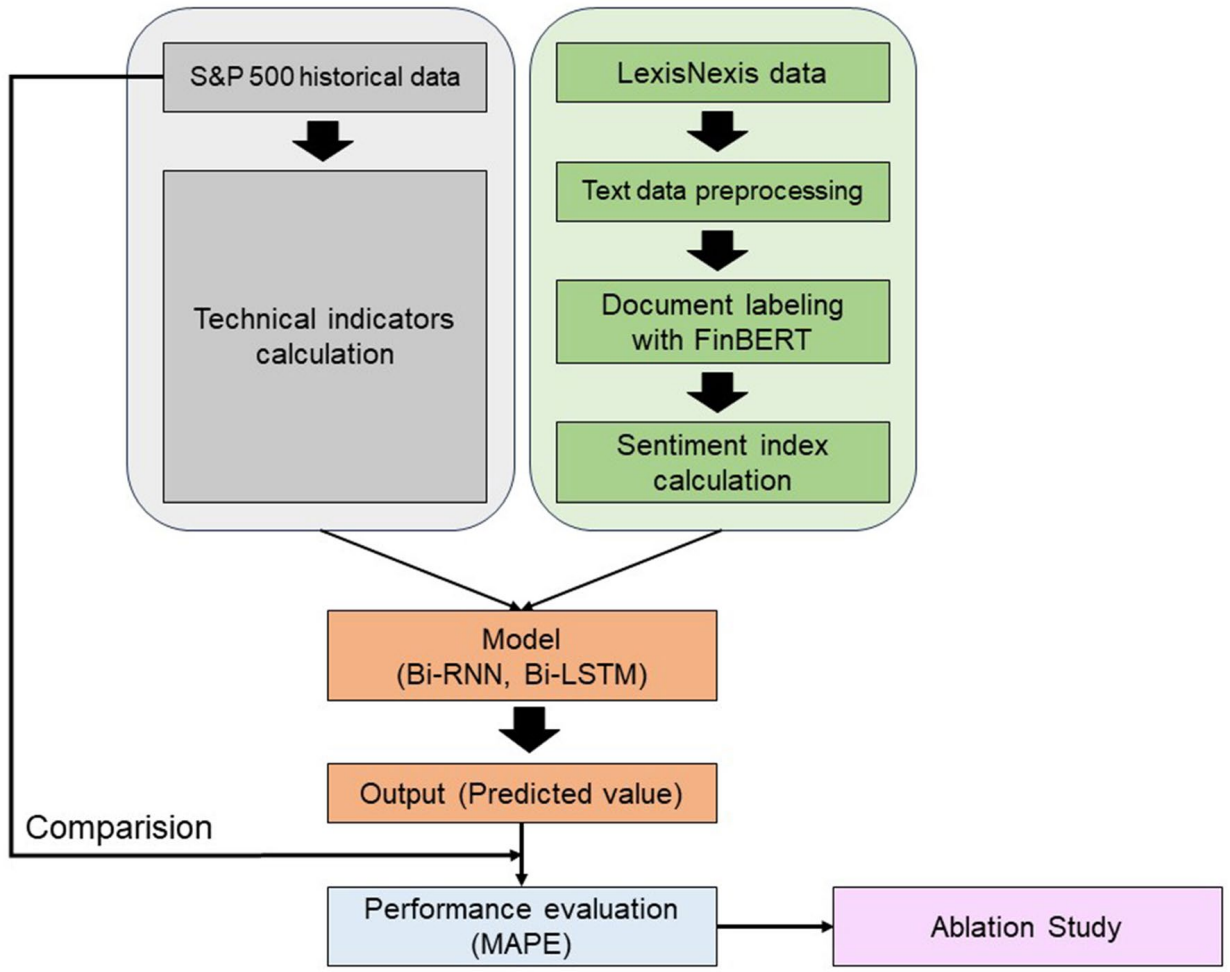
Flowchart for predicting S&P 500 index.

This section describes the experimental flow. First, data were collected for the experiment. Subsequently, preprocessing was performed to eliminate irrelevant textual data. Third, technical indicators were derived from the S&P 500 dataset, with sentiment scores generated from ESG-related news data. After combining the processed data, the scaled data were adjusted as input data for the deep learning models to forecast future prices. Lastly, MAPE was employed as the assessment measure for regression performance. In addition, ablation tests were performed to evaluate the effectiveness of each input feature. The experimental procedure is illustrated in Fig. 1.

### Data collection

The S&P 500 index is used to grasp and monitor the overall trends of the stock market and is considered one of the indicators representing the health of the United States’ financial markets^26^. The S&P 500 represents an index 500 major U.S. companies, it reflects market-wide movements rather than individual company stock prices. In addition, the S&P 500 includes companies from a variety of industries and sectors. Therefore, constructing a stock price prediction model including data from various industries is equivalent to designing a generalized model with versatility. Moreover, while stocks of individual companies must also consider the influence of internal factors, the S&P 500 is influenced by the overall market perception^27^. Consequently, building an enhanced stock price prediction model by integrating ESG information and the S&P 500 can underscore the significance and impact of sustainability information across the market to investors and relevant researchers.

The experiments were conducted by gathering two datasets spanning from January 1, 2016, to July 31, 2023. Through LexisNexis, the authors accessed and collected a collection of 14,049 news articles using the search term "ESG." Access to the LexisNexis database may require a paid subscription, such as institutional access. Additionally, historical data on the S&P 500 index, containing information such as date, closing value, opening value, high value, low value, trading volume, and volatility, for the same time periods were sourced from investing.com.

### Feature engineering

Based on previous research, the authors obtained various technical indicators that have been shown to impact stock prices using the TA-lib module^28,29^. The chosen features were opening price, closing price, high price, low price, trading volume, RSI, SMA_5, SMA_20, EMA, MACD, signal, Stochastic RSI_fastk, Stochastic RSI_fastd, Stochastic Oscillator Index_slowk, Stochastic Oscillator Index_slowd, stochastic oscillator index_slowd, WilliamR, Momentum, and ROC. Detailed descriptions of these technical indicators are provided below.

The opening price is the price of a stock at the beginning of a trading session and indicates the first transaction made for the day. High prices represent the highest value of a stock trade within a specific trading period, whereas low prices signify the lowest. Trading volume, which reflects market activity, is the number of shares or contracts traded during a specific period.

The RSI is a momentum oscillator that measures the speed and change in price movements and helps identify overbought or oversold conditions. SMAs are average closing prices over a specified number of periods. For instance, SMA_5 and SMA_20 represent the 5-day and 20-day moving averages, respectively. The EMA responds better to recent price changes by assigning more weight to them^30^.

MACD is a momentum indicator that follows trends by illustrating the interaction between two moving averages of a security’s price. Signal lines, i.e., the moving averages derived from MACD lines, play an important role in generating valuable buy-and-sell signals for traders and investors^31^.

Stochastic RSI_fastk and Stochastic RSI_fastd computed based on both the RSI and stochastic oscillator effectively grasp potential points of price reversal and enhance the accuracy of predictions^32^. To ensure smoothness, the stochastic oscillator indices_slowk and stochastic oscillator indices_slowd were considered supplementary components of the stochastic oscillator.

Another integral aspect of the analysis was William’s %R, commonly referred to as Williams R. This momentum indicator assesses whether market conditions indicate overbought or oversold scenarios, thereby contributing to a comprehensive understanding of market sentiment^33^.

Next indicators employed is momentum. The concept of momentum can be used to measure the rate of price change. Momentum provides insights into the rate at which prices change by quantifying the rate of change in stock prices. Finally, the ROC, a metric similar to momentum, involves calculating changes in prices over a specific period, providing insights into the extent of price fluctuations^34^.

### Sentiment index calculation using financial bidirectional encoder representations from transformers (FinBERT)

Preprocessing including stopwords removal and lemmatization was conducted on the news data, followed by sentiment analysis using FinBERT. FinBERT is built upon the BERT architecture, which is an effective language model for natural language processing and understanding by encoding text by considering context bidirectionally^35^. FinBERT specializes in domain knowledge by retraining BERT’s pretrained model with financial data. FinBERT takes financial-related texts such as financial news, reports, and web posts as inputs, and analyses and predicts the sentiment of the text, categorizing it as either positive, negative, or neutral.

The scores in the data were labeled 0 for negative sentiments and 1 for positive sentiments (Eq. (1)). Referring to a study by Wu et al.^36^, sentiment measurements were calculated as the difference between the number of negative and positive posts in a specific dataset.

1 $$Sentiment\, score=\frac{{M}_{tpos}-{M}_{tneg}}{{M}_{tpos}+{M}_{tneg}}$$

where ${M}_{tpos}$ represents the number of positive news articles and ${M}_{tneg}$ represents the number of negative articles on day t. The range of values for the sentiment index was between −1 and 1^25^. If the sentiment index value approaches −1, it suggests a negative tone in the news for that date. Conversely, if it approaches 1, it indicates an overall positive tone in the news. Before employing the selected features as input to the framework, a min–max scaler was applied to standardize the range of these values between 0 and 1.

### Window size

**Figure 2:**
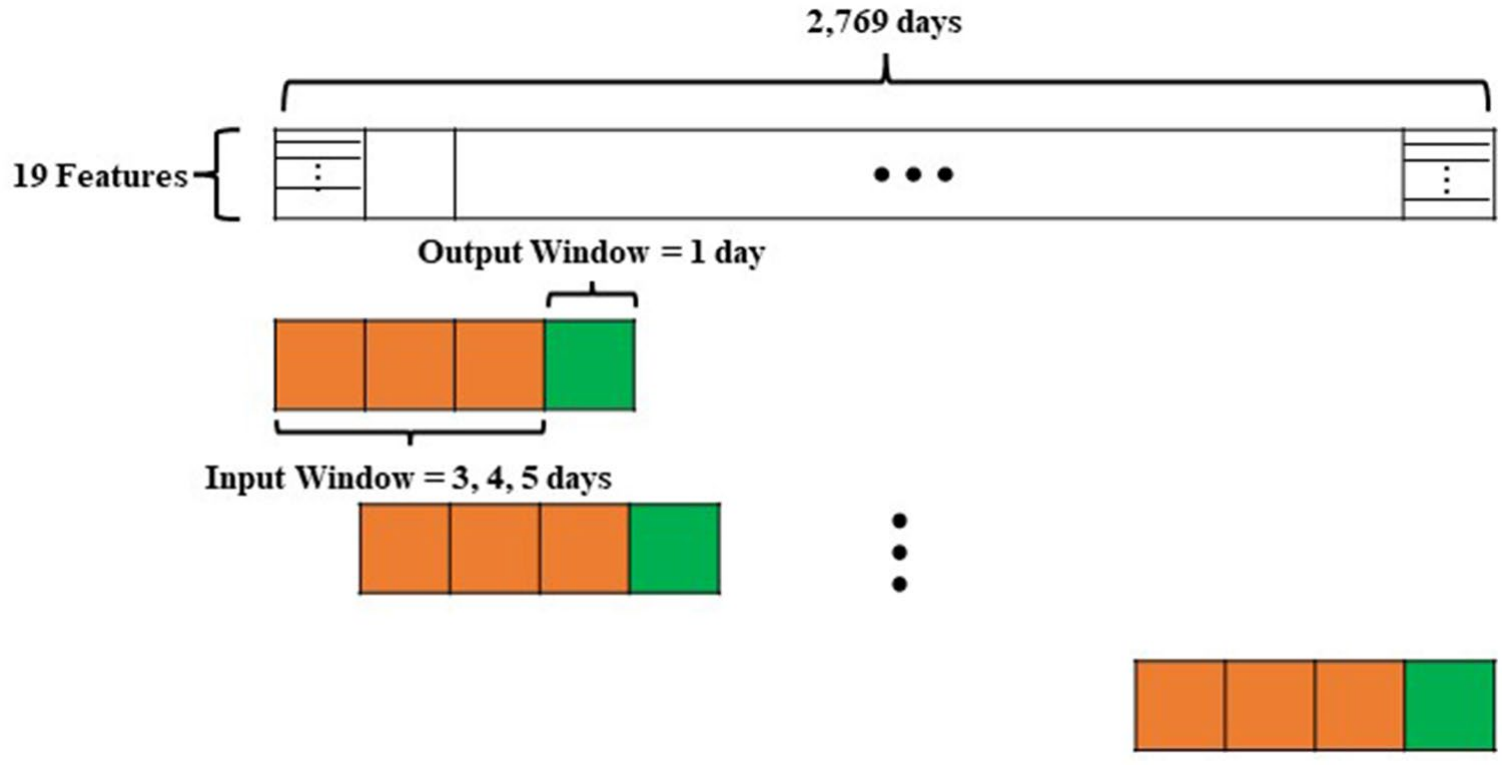
Window size illustration.

Subsequently, multiple datasets are generated, each corresponding to a distinct hyperparameter window. Window size is a fundamental concept in stock price predictions for processing and predicting time-series data^37,38^. The window size defines a fixed unit period, with the data within this window used to predict future stock prices. Therefore, selecting an appropriate window size is crucial to improving the performance of stock price prediction models. In this study, experiments were conducted using three window sizes: 3, 4, and 5 (Fig. 2). Finally, the training and test datasets were split at an 8:2 ratio. The validation dataset comprises 20% of the training dataset.

### Deep learning models

Bidirectional recurrent neural networks (Bi-RNN) are a type of recurrent neural network capable of considering both the preceding and subsequent contexts of a sequence. This bidirectional characteristic enables them to capture patterns in different temporal directions^39^. Moreover, since short-term factors can influence the fluctuation in stock prices, the RNN structure with recurrent layers is adept at capturing these changes, rendering it suitable for application as a time series model. Additionally, Bi-RNN has a flexible structure that can be applied to various types of time series data, making it useful for processing patterns. By contrast, bidirectional long short-term memory networks (Bi-LSTM) represent an enhanced iteration of RNNs that incorporate LSTM cells^40^. They excel at learning long-range dependencies and are particularly effective in tasks involving sequential data, such as time-series forecasting^41^.

## Results

The results of this study were obtained by conducting experiments using various combinations of window sizes (3, 4, and 5) and parameters, with batch sizes considered as combinations of 2, 4, 6, and 8; hidden sizes were set at 32 and 64; layer numbers at 4, 6, and 8; the number of epochs was fixed at 10 to explore all possible scenarios. The models used were Bi-RNN and Bi-LSTM.

Performance was evaluated using the MAPE, calculated using Eq. (2):

2 $$MAPE= \frac{1}{n}\sum_{t=1}^{n}|\frac{{A}_{t}-{F}_{t}}{{A}_{t}}|\times 100$$

where ${A}_{t}$ is the actual value, ${F}_{t}$ is the predicted value at time t, and n is the total number of observations. The MAPE value ranges from 0 to 100%, with values closer to 0% indicating more accurate predictions by the model^42^.

**Figure 3:**
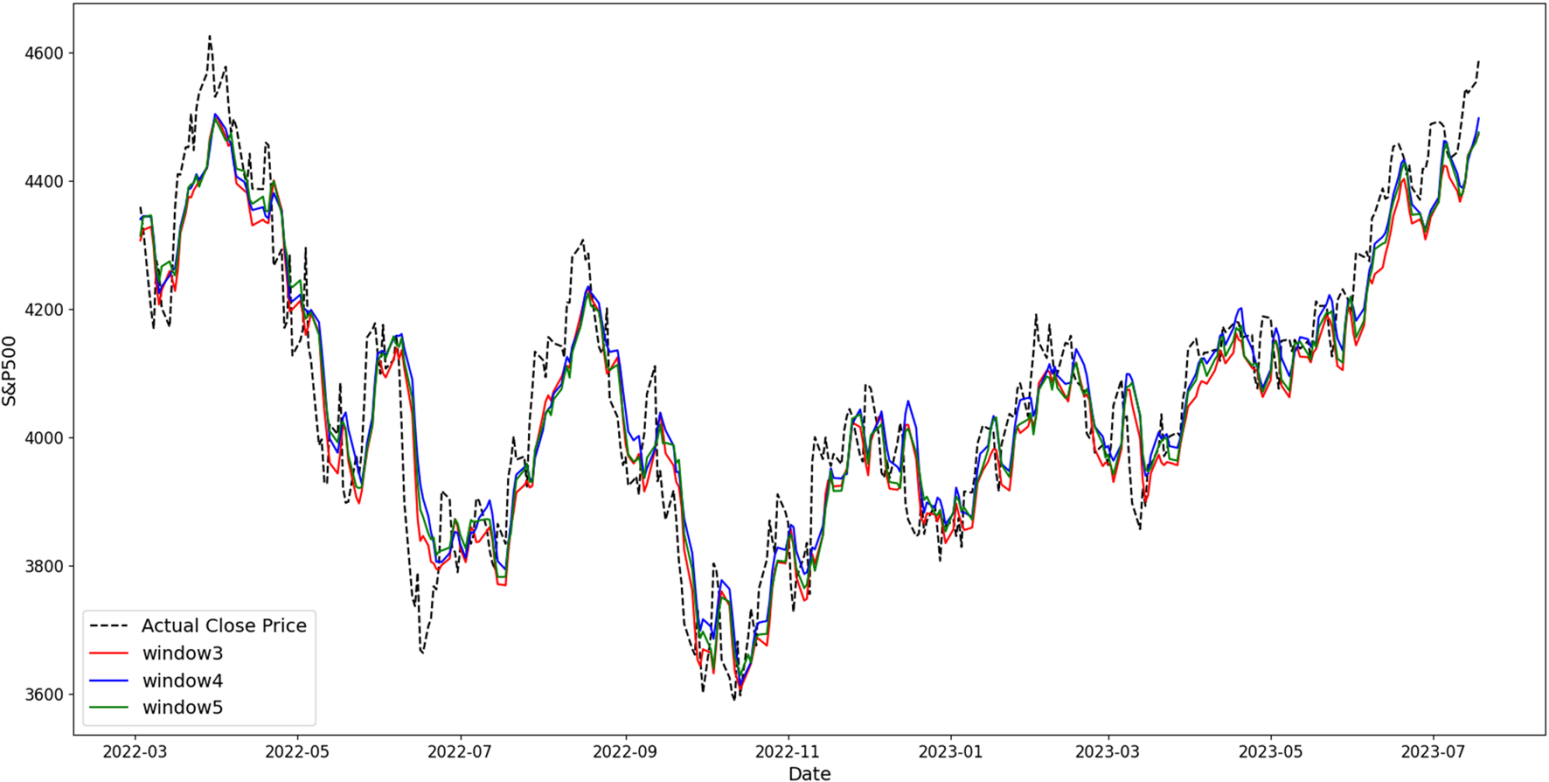
Comparison of Bi-LSTM results based on window size with the actual S&P 500 closing value.

Consequently, with a window size of 3, batch size of 64, hidden sizes of 64 and 32, and layer count of 2, the Bi-LSTM model exhibited the highest performance with a MAPE value of 3.05% on the test data (Table 2). Additionally, to convert and visualize the range of actual values of the S&P 500, an inverse transformation was performed for each window size in the Bi-LSTM model, and the results were compared (Fig. 3).

**Table 2:** Results of each regressor (MAPE, %).

Window size	Model	
	Bi-RNN	Bi-LSTM
3	4.65	3.05
4	6.85	3.2
5	5.07	3.55

**Figure 4:**
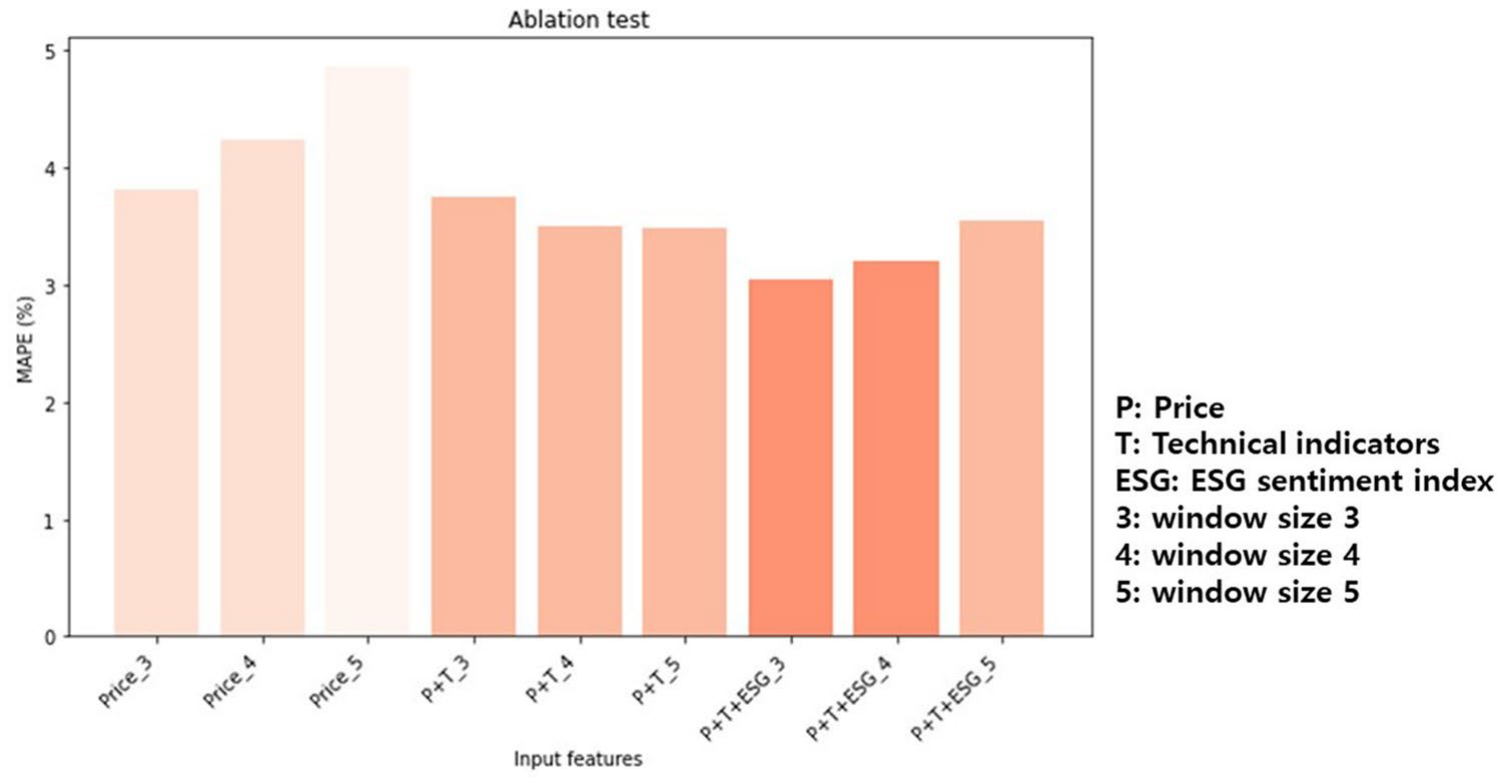
Visualization of the ablation test results.

Subsequently, an ablation test was conducted to validate the effectiveness of the input features. The ablation test is a method used to investigate causation. This method tests specific elements or variables by removing them to see how they affect the system^43^. The authors fixed the window size to 3, 4, and 5 and divided the tests into three cases (i.e., ‘only price,’ ‘price and technical indicators,’ ‘price, technical indicators, and ESG sentiment index.’). The findings revealed that combining ESG sentiment and technical and price data resulted in superior performance compared to relying solely on price data. In conclusion, the optimal performance of the Bi-LSTM model was achieved when all three inputs were integrated. These findings validate through ablation testing that there was a causal relationship between the predictive model performance of the S&P 500 index and ESG information. The specific outcomes of the MAPE values are outlined in Table 3, with a visual representation of these results shown in Fig. 4.

**Table 3:** Ablation test results based on different input features (MAPE, %).

Input features	Bi-LSTM
Only Price (window size = 3)	3.81
Only Price (window size = 4)	4.24
Only Price (window size = 5)	4.87
Price and technical indicators (window size = 3)	3.75
Price and technical indicators (window size = 4)	3.51
Price and technical indicators (window size = 5)	3.48
Price, technical indicators, and ESG sentiment index (window size = 3)	3.05
Price, technical indicators, and ESG sentiment index (window size = 4)	3.2
Price, technical indicators, and ESG sentiment index (window size = 5)	3.55

## Discussion

Sustainability has emerged as a significant global trend that shapes the landscape of modern businesses, requiring the integration of ESG metrics to evaluate corporate performance. ESG metrics provide valuable insights into operational practices and significantly affect investor trust and decision-making when a company embraces sustainable practices and effective governance. The increasing connection between sustainability, ESG metrics, and investor choices underscores the significance of incorporating ESG criteria into financial assessments, thereby merging corporate responsibility with investment strategies^1,2^. With this understanding, companies are striving to adopt strategies that enhance social responsibility and environmental impact by integrating ESG information, and these efforts can be reflected to the public through news articles. The exposure through such media can ultimately influence corporate value and, consequently, stock prices.

With the evolution of financial markets adapting to these changes, integrating ESG indicators to forecast the S&P 500 index represents a forward-thinking strategy aligned with these evolving trends^6^. Moreover, integrating fundamental technical indicators for price trend analysis with ESG indicators constitutes a comprehensive approach that significantly consider both long-term price movements and short-term price trends.

In addition, the application of machine learning in previous studies has not fully utilized the potential of leveraging advanced algorithms to comprehensively analyze the interaction of ESG metrics and technical indicators for S&P 500 prediction, indicating that previous studies may not have thoroughly explored the potential offered by machine learning.

To overcome these limitations, the authors applied a deep learning model to sentiment scores obtained by applying FinBERT to LexisNexis news data and 18 technical indicators acquired from historical data of S&P 500 index. Subsequently, the MAPE was used as the performance evaluation metric for the framework. After undergoing cross-validation with various parameters, the Bi-LSTM model demonstrated a superior MAPE of 3.05 on the test dataset when employing a window size of 3, batch size of 64, hidden size of 32 and 64, and a layer count of 2. Moreover, ablation tests conducted in this study demonstrated the strength of the selected input features for the S&P 500 index prediction. Specifically, considering a sentiment index incorporating ESG information alongside technical indicators and price information yielded the best performance.

Consequently, integrating ESG metrics and technical indicators to predict the S&P 500 index has significant practical implications. As ESG criteria emerge as favorable factors for stock predictions, they drive companies to evaluate their operational practices and sustainability efforts. Moreover, investors who recognize the influence of ESG metrics can make informed decisions by trusting companies that prioritize sustainability and effective governance. This interaction among sustainability considerations, ESG metrics, and investor choices highlights the need to integrate ESG elements into financial assessments and align corporate responsibility with investment strategies.

Furthermore, this study aligns with financial market trends and demonstrates the potential of combining ESG and technical indicators to predict stock market behavior. Deep learning models provide innovative opportunities for comprehensively examining the intricate connections between ESG and technical indicators, leading to precise S&P 500 forecasts.

## Limitations of the study

This study has several limitations. First, the findings may not comprehensively represent the intricate dynamics of the entire financial market due to their reliance on the S&P 500 dataset. Future research should validate and extend these results by incorporating data from a wider spectrum of financial markets. Second, the sentiment index used in this analysis was derived solely from news data, which is a potential limitation. Therefore, exploring the integration of diverse textual data related to ESG could help conduct a more comprehensive and robust analysis. Third, the value of focusing on ESG is different depending on the industry. Therefore, future research could attempt to predict stocks by industry group rather than the entire stock market.

## Data Availability

No datasets were generated or analysed during the current study.
